# An extended release local anaesthetic: potential for future use in veterinary surgical patients?

**DOI:** 10.1002/vms3.43

**Published:** 2016-08-23

**Authors:** B. Duncan X. Lascelles, Kristin Kirkby Shaw

**Affiliations:** ^1^ Comparative Pain Research Program Department of Clinical Sciences College of Veterinary Medicine North Carolina State University Raleigh North Carolina; ^2^ Comparative Medicine Institute and Department of Clinical Sciences College of Veterinary Medicine North Carolina State University Raleigh North Carolina; ^3^ Center for Pain Research and Innovation UNC School of Dentistry Chapel Hill North Carolina; ^4^ Animal Surgical Clinic of Seattle 14810 15th Ave NE Shoreline Washington 98155

**Keywords:** bupivacaine liposome injectable suspension, pain, perioperative, dog

## Abstract

One of the most effective means of preventing the transduction and transmission of acute and perioperative pain is through the use of local anaesthetics. However, local anaesthetics currently available have a relatively short duration of action. Although there are several tools available to treat perioperative pain in companion animals, overall, there is an unmet need for products that can be administered in the clinic, and provide pain relief for the crucial first few days following surgery in the home environment. Specifically, in relation to local anaesthetics, there is a clear unmet need for a long‐acting local anaesthetic that can be added to the multimodal analgesic protocol to provide pain relief to patients in the home environment or during extended hospitalization. Bupivacaine liposomal injectable suspension recently became available for use in humans, and has proven efficacious and safe. This paper will review the use of local anaesthetics, particularly bupivacaine, in dogs and cats, and introduce a new formulation of prolonged release bupivacaine that is in development for dogs and cats.

All surgical procedures result in some degree of tissue trauma and associated pain. Over the last two decades in veterinary medicine, there has been a dramatic shift in the approach to perioperative pain, with an increased recognition of pain and increased use of analgesics to control perioperative pain (Lascelles & Capner 1999; Hunt *et al*. 2015). Most would agree that as veterinary surgeons, it is our ethical obligation to minimize pain and suffering in animals, and it is recognized that pain delays healing and return to function and can result in chronic postoperative pain. In humans, interest in chronic pain after surgery has dramatically increased since the finding that more than 20% of 5000 of patients attending chronic pain clinics cited surgery as the cause for their chronic pain (Crombie *et al*. 1998) and it appears that even common minor procedures such as hernia repair have a significant risk of chronic pain. Unmanaged acute pain can lead to chronic, maladaptive pain through the process of neuroplasticity, or remodelling of pain pathways (Reddi & Curran 2014). Chronic, maladaptive pain is very difficult to manage, whereas post‐surgical pain is generally considered easier to manage (Grichnik & Ferrante 1991; Woolf 2010).

In the authors' opinion, there are four central tenets to optimizing perioperative analgesia: (i) provide pre‐emptive analgesia, (ii) utilize multimodal pain management, (iii) deliver overlapping/ continuous analgesia and (iv) match the analgesic plan to the degree of surgery. The pain transmission system is complex, with a lot of redundancy, and therefore in order to minimize postoperative pain and any long‐term sequelae of unrelieved pain, an effective multimodal approach should be employed that interrupts the pain transmission and detection system at multiple levels (Fig. 1). Veterinarians should consider methods of simultaneously minimizing the transduction and transmission of pain in peripheral tissue, modulation of pain in the spinal cord and the conscious perception of pain using drug and non‐drug approaches (Fig. 1).

**Figure 1:**
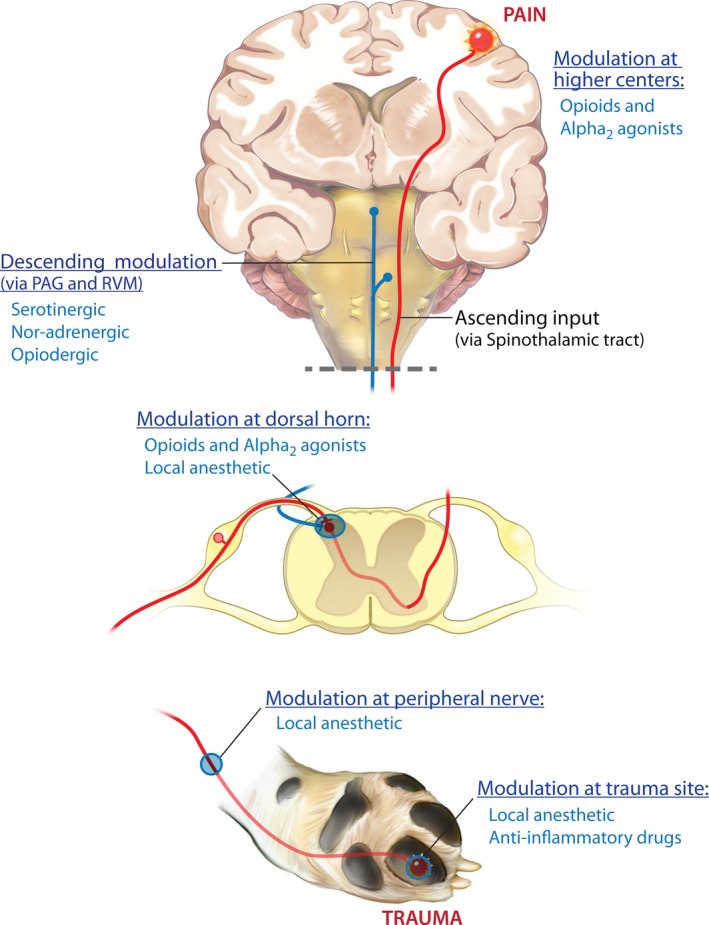
Sites of drug modulation of the nociceptive transmission pathway. Primary afferent neurons carry nociceptive information into the dorsal horn of the spinal cord, where they project (in a complex manner) onto second‐order neurons. Projection (second order) neurons from the dorsal horn transmit information to the somatosensory cortex via connections in the thalamus. This information encodes information about the location and intensity of pain. Other projection neurons, via connections in the brainstem (parabrachial nucleus) and amygdala, transmit information to the cingulated and insular cortices and encode information about the affective component of pain. Ascending projection neurons also connect with neurons in the periaqueductal grey (PAG) and rostral ventral medulla (RVM), as do neurons from higher centres, and neurons from the RVM send descending information that regulates nociceptive output from the spinal cord. This nociceptive transmission and pain detection system is complex, and has a lot of redundancy, and so the most effective way to dampen down or prevent signals moving through the system is to use ‘multimodal analgesia’, or ‘balanced analgesia’. This is the concept of simultaneously using different classes of analgesic drugs (e.g. local anaesthetics and non‐steroidal anti‐inflammatory drugs) that act on different parts of the nociceptive transmission pathway to improve efficacy and decrease side effects seen when large doses of individual drugs are used. Of all the analgesic drug classes, only local anaesthetics have the potential to completely block all nociceptive signals.

There are few data as to how long post‐surgical pain persists in animals, and this time period will vary with the type of surgical procedure performed (Tomas *et al*. 2015). The inflammatory phase of wound healing typically lasts approximately 72 h (Leaper and Harding 1998), and this is the period that has been recommended as the minimum amount of time analgesics should be provided following surgery. Post‐surgical pain can generally be well controlled while an animal is hospitalized using injectable opioids, ketamine, cyclooxygenase inhibiting NSAIDs and local anaesthetics. However, most veterinary patients that undergo elective soft tissue or orthopaedic surgery are discharged from the veterinary hospital the same day, or within 24 h following surgery. There is a need to bridge between analgesics provided in the hospital and effective pain relief in the home environment. Currently in the United States, only oral cyclooxygenase inhibiting NSAIDs and transdermal fentanyl liquid (Recuvyra^™^, Elanco) have been approved for post‐surgical analgesia in dogs, and injectable buprenorphine (Simadol^™^, Zoetis) and the cyclooxygenase inhibiting NSAID robenicoxib (Onsior^™^, Elanco) in cats. Meloxicam (Metacam^™^, Boehringer Ingelheim) is approved for use in cats preoperatively, but only for a single dose. Beyond these options, there are unapproved fentanyl patches manufactured for human use, which have been suggested to be efficacious (Kyles *et al*. 1998), but there are legal considerations associated with sending a non‐approved scheduled drug into the client's home, and unfortunately child deaths have resulted from access to fentanyl patches (Teske *et al*. 2007).

One of the most effective means of preventing the transduction and transmission of pain is through the use of local anaesthetics. Indeed, it could be argued that the only available analgesics that can completely block perioperative pain are the local anaesthetics. It is interesting therefore that local anaesthetics appear to be one of the least used classes of analgesic in small animal practice (Hunt *et al*. 2015). Recommended methods of providing local anaesthetics include wound/tissue infiltration, regional nerve blocks, neuraxial analgesia (intrathecal, epidural) and the placement of soaker catheters (Table 1) (Mathews *et al*. 2014). While these techniques are relatively well described in textbooks, there are a number of factors that may contribute to the documented low frequency of use of local anaesthetics on a routine basis, compared to other analgesics (Hunt *et al*. 2015). Veterinarians perceive that there is technical difficulty associated with nerve blocks, and much of this probably stems from the poor descriptions that exist for many nerve blocks in veterinary textbooks and journal publications. For example, only recently was the feline distal forelimb nerve block described in sufficient detail in the literature to allow for accurately performing this block (Enomoto *et al*. 2015). Other barriers to the use of local anaesthetics in practice may be the relatively short duration of action of available local anaesthetic formulations (Table 2). The placement of fenestrated wound catheters and the intermittent administration of local anaesthetic through the catheters has been described as a technique to extend the duration of analgesia obtained from local anaesthetics (Davis *et al*. 2007). In our hands, these have been very effective, but do require the placement and maintenance of a fenestrated catheter into the wound of *hospitalized* patients.

**Table 1:** Methods of using local anaesthetics in veterinary medicine

Topical application
Local infiltration (non‐specific) using single or multiple doses
Continuous infiltration blocks with fenestrated catheters
Regional nerve blocks
Brachial plexus block
Neuraxial blocks
Epidural (single injection, catheter)
Intrathecal (single injection, catheter)
Selective nerve blocks
Aural
Eye and orbit
Dental (maxillary, mandibular)
Paravertebral block
Selective block of radial, median, ulnar and musculocutaneous nerves
Mid‐humeral
Distal branches
Selective block of femoral (saphenous) and sciatic (common peroneal, tibial) nerves
Intercostal nerve blocks
Intra‐cavity
Intra‐thoracic
Intra‐peritoneal
Intravenous local anaesthetics
Intravenous regional anaesthesia (‘Bier block’)
Systemic (treatment of postoperative and neuropathic pain)

**Table 2:** Recommended doses and expected onset time and duration of action for single doses of commonly used local anaesthetics in cats and dogs

Drug	Single injection (dog)	Single injection (cat)	Time to onset	Duration of action
Lidocaine	4 mg/kg	2 mg/kg	<5 min	Up to 2 h
Bupivacaine	2 mg/kg	2 mg/kg	10–15 min	Up to 8 h

Overall, there is a clear unmet need for effective analgesic products that can be given in the clinic and continued in the home environment, or products that can be administered in the clinic and provide pain relief for the crucial first few days following surgery in the home environment. Specifically, in relation to local anaesthetics, there is a clear unmet need for a long‐acting local anaesthetic that can be added to the multimodal analgesic protocol to provide pain relief to patients in the home environment or during extended hospitalization. Bupivacaine liposomal injectable suspension is currently being developed for dogs and cats, with a target indication of 72 h of local analgesic efficacy following intra‐operative tissue infiltration (http://www.aratana.com/therapeutics/pipeline/pain/, accessed 23 October 2015). This paper will review the use of local anaesthetics, particularly bupivacaine, in dogs and cats, introduce a new formulation of prolonged release bupivacaine and review the published literature on this product.

## Local anaesthetics

### Mechanism of action

The mechanism of action of local anaesthetics have been reviewed in detail elsewhere (Lirk *et al*. 2014), but briefly, they block cell membrane sodium channels on neurons thus preventing the propagation of action potentials and transmission of nociceptive signals. Sodium channel block is brought about by a conformational change and the creation of a positive charge in the channel pore after the local anaesthetic has reached the local anaesthetic binding site in the axon either from the cytoplasmic compartment (classic hydrophilic pathway) or directly via its lipid membrane (hydrophobic pathway) or via large‐pore channels (alternative hydrophilic pathway). Local anaesthetics differ in their chemical structures and can broadly be categorized into amides (lidocaine, bupivacaine, mepivacaine) and esters (procaine, tetracaine). The chemical structure influences the solubility and metabolism of the drug. The two most commonly used local anaesthetics in veterinary medicine are lidocaine (rapid onset of action; 1–2 h duration of action) and bupivacaine (slower onset of action; up to 8 h duration unless formulated for prolonged release). Both drugs are metabolized by the liver. Work performed in dogs (*n* = 6 for each group) reported the average dose and arterial plasma concentration at seizure onset for several local anaesthetics (Feldman *et al*. 1989). Seizures occurred at doses of 20.8 ± 4.0 mg/kg IV and 47 200 ± 5400 ng/mL arterial plasma concentration for lidocaine, and a dose of 4.31 ± 0.36 mg/kg and arterial concentration of 18 000 ± 2700 ng/mL for bupivacaine. The authors also concluded that on a mg/kg basis, bupivacaine was more arrhythmogenic than lidocaine (Feldman *et al*. 1989). An intravenous dose of 4 mg/kg of bupivacaine produced cardiac electrophysiological abnormalities and cardiovascular depression, reducing measures of left ventricular pressure by 50% (Bruelle *et al*. 1996). In a study evaluating the effects of different plasma concentrations of bupivacaine following infusion, plasma concentrations up to 1250 ng/mL were not associated with any cardiovascular depression, but levels of 2500 ng/mL and greater were (Fujita 1994 Jun).

### Effects on tissue

The effects of local anaesthetics on wound healing have been investigated in many *in vitro* and *in vivo* models. While there is some evidence that these drugs alter the cellular events of early tissue healing, there does not appear to be a clinically significant impact on wound healing or mechanical wound strength in preclinical animal studies (Abrão *et al*. 2014) or humans. Probably the most feared complication of the use of local anaesthetics is the fear of wound infection. This appears to be a theoretical risk since there are no data from published studies to justify this fear. A review of studies evaluating the use of analgesic catheters and continuous local anaesthetic installation into wounds found that reported wound infection rates were similar between active (0.7%) and control groups (1.2%) (Liu *et al*. 2006). Furthermore, local anaesthetics have well‐documented bacteriostatic and bactericidal actions (Sakuragi *et al*. 1996, 1998; Johnson *et al*. 2008). Several studies have found that bupivacaine (concentrations between 0.125% and 0.75%) is able to inhibit the growth of pathogenic bacteria and fungi, including *Escherichia coli*,* Staphylococcus aureus*,* Staphylococcus epidermidis*,* Candida albicans* and others (Sakuragi *et al*. 1996, 1998; Johnson *et al*. 2008).

Another complication that may be raised is the possibility of seroma formation; however, this is not a recognized complication with recommended doses, even repeated doses, of currently used local anaesthetics. However, bupivacaine and other local anaesthetics have demonstrated chondrotoxicity, particularly when delivered in high concentration or with extended duration of exposure to compromised cartilage. The implication of a single intra‐articular injection of local anaesthetic, as may be performed at the time of orthopaedic surgery, is unclear. However, current opinion is that prolonged, high doses of intra‐articular local anaesthetics should not be used, and single lower dose administrations should be used intra‐articularly with caution (Scott 2010; Piper *et al*. 2011).

### Use in canine and feline patients

Local anaesthetics are widely available in veterinary small animal practice and have been shown to provide enhanced, multimodal analgesia with little risk for untoward effects. The World Small Animal Veterinary Association Global Pain Council (WSAVA GPC) strongly recommends the routine use of local anaesthetic (Mathews *et al*. 2014), and these recommendations were echoed in the 2015 pain management guidelines from the American Animal Hospital Association (Epstein *et al*. 2015).

One of the most straight‐forward ways to employ the use of local anaesthetics is through incisional block, either preoperatively or at the time of wound closure, and this technique has been advocated as a means of enhancing multimodal perioperative pain management (Scott 2010). Bupivacaine can be instilled through a 22–25 g needle into the subcutaneous tissue along the incisional line and is expected to provide several hours of analgesia postoperatively. If extended duration of analgesia is desired, a wound ‘soaker catheter’ may be placed (Davis *et al*. 2007). Repeated administration of local anaesthetic through this catheter can provide extended analgesia throughout the hospitalization period (Hardie *et al*. 2011), and regardless of the catheter type used, bolus administration is thought to provide for better dispersion of local anaesthetic throughout the wound than continuous infiltration (Hansen *et al*. 2013). Studies in human patients have highlighted the importance of where the analgesic catheter is placed within the wound (Wu *et al*. 2005), suggesting that placement within the muscle in a surgical wound is important. One concern with this is that intra‐muscular injection of local anaesthetics reliably produces myotoxicity in experimental studies, however a recent review concluded that this was not a problem in the clinical setting (Zink 2004).

Local anaesthetics are available in patches for dermal application, indicated for neuropathic pain in humans. Penetration depth, even with the new ‘heated’ patches is only about 8 mm (Wallace *et al*. 2010), and analysis of several studies suggests there is not a measurable analgesic effect associated with their use on surgical wounds (Bai *et al*. 2015).

Local anaesthetics are also frequently used for regional nerve blocks and these techniques have demonstrated significant enhancement of postoperative analgesia in dogs and cats (Campoy *et al*. 2012; Aguiar *et al*. 2014). However, the duration of analgesia using these techniques is also limited due to the duration of action of the currently available formulations, as shown nicely in experimental studies (Trumpatori *et al*. 2010). Recently, the first FDA‐approved long‐acting preparation of bupivacaine for use in human medicine has become available, a bupivacaine liposomal injectable suspension (Pacira Pharmaceuticals, Inc., Parsippany, NJ) and other preparations appear to be in development (http://www.durect.com/wt/durect/page_name/postop).

## Bupivacaine liposomal injectable suspension

**Figure 2:**
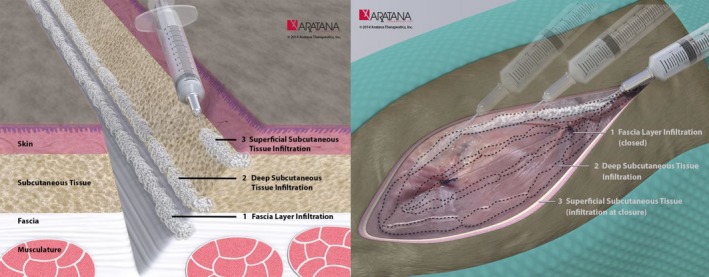
The recommended method for injection of bupivacaine liposomal injectable suspension into tissues is illustrated in these images produced by Aratana Therapeutics (http://www.aratana.com/therapeutics/pipeline/pain/, accessed 23 October 2015). All layers of the tissue in the area to be blocked should be infiltrated with the bupivacaine liposomal injectable suspension. Although the free bupivacaine released following breakdown of the lipid bilayers will diffuse just as regular bupivacaine does, the liposome particles diffuse less readily through tissue, and hence the need to carefully distribute the preparation throughout the wound. To do this, all layers of the wound are infiltrated using a moving needle technique, where the needle is inserted into the tissues, aspiration is performed to ensure the end of the needle is not within a vessel and the suspension is injected as the needle is withdrawn. This is repeated in all layers and at all parts of the wound, using the calculated dose and volume.

In 2011, the FDA approved a prolonged‐release formulation of bupivacaine, bupivacaine liposome injectable suspension (Pacira Pharmaceuticals, Inc., Parsippany, NJ) for use as a single‐dose infiltration into the surgical site to effect post‐surgical analgesia in human surgical patients. The prolonged‐release technology used in this product consists of multi‐vesicular liposomes encapsulating aqueous bupivacaine. The liposomes are microscopic structures made of non‐concentric lipid bilayers designed such that bupivacaine is gradually released from vesicles over 96 h as the lipid bilayers break down. The lipids making up the bilayer structures consist of phospholipids, cholesterol and triglycerides, and importantly do not contain lecithin which has been associated with tissue necrosis and toxicity. The technique for instilling bupivacaine liposomal injectable suspension into a wound differs slightly from using traditional bupivacaine formulation because the liposomes do not readily diffuse into tissues. This is due to the size of the liposomes – each liposome is approximately 10–30 μm in diameter (for reference, a canine red blood cell is approximately 6–8 μm in diameter). A moving needle technique is used to inject the solution into all tissue layers within the surgical field (Fig. 2). As bupivacaine is gradually released from individual liposomes, it distributes locally to the surrounding tissues and eventually into the systemic circulation.

### Studies in humans

As part of the FDA approval process, two multi‐centre, randomized, double‐blind, placebo‐controlled trials were performed in human surgical patients to evaluate efficacy. One study compared bupivacaine liposome injectable suspension to a saline placebo in human patients undergoing hemorrhoidectomy and found significantly lower pain scores in the group receiving bupivacaine liposome injectable suspension compared to placebo over the first 72 h (*P* < 0.0001). The 30% reduction in pain compared to placebo over the 72 h was considered clinically meaningful. Furthermore, bupivacaine liposome injectable suspension‐treated patients required significantly less opioid medication (*P* < 0.0008) during the first 72 h and had significantly greater overall satisfaction with postoperative analgesia (*P* = 0.0007) (Gorfine *et al*. 2011).

Another study, constituting the evaluation in orthopaedic surgery, compared bupivacaine liposome injectable suspension to saline control in human patients undergoing bunionectomy and reported significantly reduced pain scores at 24 and 36 h post‐surgery in the bupivacaine liposome injectable suspension‐treated group. In this same study, significantly fewer patients treated with bupivacaine liposome injectable suspension required rescue analgesia (Golf *et al*. 2011).

Since its approval, bupivacaine liposome injectable suspension has become widely used in human surgical patients, including those undergoing joint replacement, soft tissue reconstructive procedures and a variety of other soft tissue and orthopaedic surgeries.

Bupivacaine liposome injectable suspension has been well tolerated in human surgical patients, and a higher margin of safety compared to traditional bupivacaine has been reported (Portillo *et al*. 2014). The most common side effects noted in human patients were nausea, vomiting and constipation. There was no significant difference in cardiotoxicity between bupivacaine liposome injectable suspension and traditional bupivacaine. A review of data from 10 prospective studies using bupivacaine liposome injectable suspension or traditional bupivacaine in human patients and found no significant impact on wound or bone healing with the use of either bupivacaine formulation (Baxter *et al*. 2013). Recently, the use of bupivacaine liposome injectable suspension has begun to be investigated for nerve block (Ilfeld *et al*. 2013) with this early information suggesting that the relationship between dose and efficacy may not be linear, but an evaluation of the safety of off‐label use of bupivacaine liposome injectable suspension for peripheral nerve block appears to indicate the side‐effect profile is identical to saline (Ilfeld *et al*. 2015). Very recently, intercostal nerve block with bupivacaine liposome injectable suspension was found to be as effective as thoracic epidural anaesthesia for patients undergoing minimally invasive intra‐thoracic surgery or open thoracotomy (Rice *et al*. 2015). Interestingly, pain control was similar in both the open and minimally invasive patients for the bupivacaine liposome injectable suspension group, but pain control was significantly less effective in the open thoracotomy patients in the thoracic epidural anaesthesia group that used regular bupivacaine.

### Studies in dogs

Bupivacaine liposome injectable suspension has been extensively studied in dogs, as part of the development of bupivacaine liposome injectable suspension for human use. These studies have centred on the local and systemic safety and tolerability of the drug following tissue infiltration. In a dog model of inguinal hernia repair, bupivacaine liposome injectable suspension was infiltrated into the surgical site at doses ranging from 9 to 25 mg/kg and a mild granulomatous inflammatory response was seen histologically, that had not resolved by 2 weeks following infiltration, but was not considered indicative of any adverse effect on wound healing (Richard *et al*. 2011a,b) and the findings were similar to control‐treated animals. For perspective, the currently approved maximal dose of bupivacaine liposome injectable suspension in humans is 3.8 mg/kg (assuming an average person weight of 70 kg). The same group also investigated the effects of repeated doses of bupivacaine liposome injectable suspension in a dog model (Richard *et al*. 2011a,b). Dogs were injected twice weekly for 4 weeks with bupivacaine liposome injectable suspension at doses of 9, 18 and 30 mg/kg, bupivacaine HCl, or saline, administered into the subcutaneous tissue over the scapulae. There were no observed clinical signs consistent with CNS toxicity and no ECG abnormalities. At days 26 and 54, there were significant differences observed histologically between bupivacaine liposome injectable suspension and the other groups, including minimal to moderate granulomatous inflammation with vacuolated macrophages, multi‐nucleated giant cells and tissue mineralization. These observations were considered a normal response to the liposomes. Rabbits were also used in these studies, and appeared to be more sensitive to the effects of this bupivacaine formulation than dogs (Richard *et al*. 2011a,b). Furthermore, the administration of a single‐dose bupivacaine liposome injectable suspension around the brachial plexus in dogs has been evaluated from a safety and neurotoxicity perspective (Richard *et al*. 2012). Doses of 9, 18 and 30 mg/kg were administered and 2 weeks later, a mild granulomatous inflammation of adipose tissue around the nerve roots was seen, but no signs of neurotoxicity were seen on haematoxylin and eosin‐stained sections. Again, no cardiovascular or central nervous system‐related adverse effects were seen. Across all these studies, plasma concentrations following injection of bupivacaine liposome injectable suspension were approximately four‐ to sixfold lower than an equivalent dose of bupivacaine solution, with a C‐max of approximately 500 ng/mL plasma, with a standard deviation of about 500 ng/mL following the 9 mg/kg dose. In humans, CNS and cardiac system adverse effects are usually seen first at >2000 ng/mL and >4000 ng/mL, and in dogs, seizures occur at 18 000 ng/mL plasma concentrations of bupivacaine (Feldman *et al*. 1989).

Bupivacaine liposome injectable suspension is currently being investigated for use in dogs and cats. At the time of writing, a multi‐centre, placebo‐controlled pilot trial in dogs undergoing orthopaedic surgery has been completed and the results presented. In dogs undergoing lateral retinacular suture stabilization of cruciate deficient stifles, 46 dogs were enrolled in a blinded, placebo‐controlled study and evaluated postoperatively using subjective assessments. Bupivacaine liposome injectable suspension was injected intra‐incisionally at the time of closure of the wound to provide postoperative analgesia. There was a significant overall treatment effect of pain reduction (*P* = 0.0027) in favour of bupivacaine liposome injectable suspension, and there were significantly more treatment successes (reduction in pain scores) in the bupivacaine liposome injectable suspension group compared to placebo over each 24‐h period (*P* = 0.0001 for 0–24 h, *P* = 0.0349 for 24–48 h and *P* = 0.0240 for 48‐72 h) up to 72 h postoperatively. No significant adverse events were seen (unpublished data). Recently, the top‐line results from the subsequently performed larger pivotal study were announced, with similar efficacy seen. Pilot studies using this product are also in progress in cats.

## Conclusion

Local anaesthetics are potentially the most effective analgesic class available to veterinarians for use in the perioperative period because they have the potential to completely block nociceptive signals from reaching the CNS. However, their clinical effectiveness is limited by the relatively short duration of action. The recent development and approval for use in humans of a prolonged duration of action liposome formulation of bupivacaine appears to be having a dramatic and positive impact in human postoperative pain control. The development of this product for the veterinary market shows promise to be an effective tool for practitioners, and would help address a significant unmet need – the ability to provide prolonged postoperative analgesia using the most effective class of perioperative analgesic available.

## Source of funding

None

## Conflicts of interest

The authors are both paid consultants of Aratana Therapeutics.

## Contribution

KKS and BDXL both planned, drafted and finalized the manuscript
